# Estimating mortality associated with seasonal influenza among adults aged 65 years and above in China from 2011 to 2016: A systematic review and model analysis

**DOI:** 10.1111/irv.13067

**Published:** 2022-11-17

**Authors:** Kaige Dong, Hui Gong, Guangjie Zhong, Xiaowei Deng, Yuyang Tian, Minghan Wang, Hongjie Yu, Juan Yang

**Affiliations:** ^1^ Shanghai Institute of Infectious Disease and Biosecurity, School of Public Health Fudan University Shanghai China; ^2^ School of Public Health, Fudan University, Key Laboratory of Public Health Safety Ministry of Education Shanghai China

**Keywords:** China, disease burden, influenza, mortality, older adults

## Abstract

**Background:**

Estimation of influenza disease burden is crucial for optimizing intervention strategies against seasonal influenza. This study aimed to estimate influenza‐associated excess respiratory and circulatory (R&C) and all‐cause (AC) mortality among older adults aged 65 years and above in mainland China from 2011 to 2016.

**Methods:**

Through a systematic review, we collected influenza‐associated excess R&C and AC mortality data of older adults aged 65 years and above for specific cities/provinces in mainland China. Generalized linear models were fitted to estimate the corresponding excess mortality for older adults by province and nationwide, accounting for the potential variables of influenza virus activity, demography, economics, meteorology, and health service. All statistical analyses were conducted using R software.

**Results:**

A total of 9154 studies were identified in English and Chinese databases, and 11 (0.1%) were included in the quantitative synthesis after excluding duplicates and screening the title, abstract, and full text. Using a generalized linear model, the estimates of annual national average influenza‐associated excess R&C and AC mortality among older adults aged 65 years and above were 111.8 (95% CI: 92.8–141.1) and 151.6 (95% CI: 127.6–179.3) per 100,000 persons, respectively. Large variations in influenza‐associated excess R&C and AC mortality among older adults were observed among 30 provinces.

**Conclusions:**

Influenza was associated with substantial excess R&C and AC mortality among older adults aged 65 years and above in China from 2011 to 2016. This analysis provides valuable evidence for the introduction of the influenza vaccine into the National Immunization Program for the elderly in China.

## 1 INTRODUCTION

The annual circulation of seasonal influenza causes a heavy disease burden on society, with 4 to 23 million lower respiratory tract infection hospitalizations and 290,000–650,000 respiratory deaths globally, of which more than half of deaths occur in the population aged 65 years and above.^1^, ^2^ In China, the burden of influenza‐associated deaths is also serious, with an average of 88,000 (95% confidence interval [CI]: 84,000–92,000) excess influenza‐associated respiratory deaths annually,^3^ 80% to 95% occurring in older adults.^3^, ^4^, ^5^, ^6^

Vaccination is a core pharmaceutical preventative intervention to protect against influenza virus infection or severe outcomes after infection. However, the influenza vaccine is not included in the National Immunization Program in China, and vaccination is paid out of pocket in the majority of regions,^7^ causing an extremely low uptake (3.8%) among older adults aged 60 years and above nationwide.^8^ As the most populous country with 191 million older adults 65 years of age or above,^9^ a reliable estimate of excess mortality associated with influenza in this population (particularly after the 2009 influenza pandemic, as the burden may change due to the displacement of seasonal A(H1N1) virus after the pandemic^3^, ^10^) is critical for policy‐making about immunization programs.

It is difficult to quantify the exact mortality burden of influenza for the following reasons: routine laboratory tests are rarely conducted across China, and secondary bacterial infections or exacerbation of existing underlying conditions triggered by influenza virus infections, can lead to death.^11^ Accordingly, influenza mortality burden studies mainly rely on applying statistical methods to estimate broadly defined disease outcomes, such as mortality from respiratory diseases, respiratory and circulatory (R&C) diseases, or all‐cause (AC) mortality.^12^, ^13^ None of these metrics could independently depict the whole picture of the mortality burden, with excess mortality attributable to respiratory diseases having the highest specificity, excess AC mortality having the highest sensitivity, and excess R&C mortality in between.^14^

To our knowledge, Li *et al* estimated the national and provincial influenza‐associated excess respiratory mortality in older adults after the 2009 influenza pandemic in mainland China.^3^ For influenza‐associated excess R&C or AC mortality, a few studies estimated mortality at the national or regional level before the 2009 influenza pandemic^4^, ^10^ or at the city level, such as Beijing,^15^ Shanghai,^16^, ^17^ and Shenzhen.^18^ To better understand the post‐pandemic influenza‐associated mortality burden among elderly individuals aged 65 years and above in mainland China, we used a systematic review and model method to estimate influenza‐associated excess R&C and AC mortality by province and nationwide from 2011 to 2016.

## 2 METHODS

Through a systematic review, we collected influenza‐associated excess R&C and AC mortality data of older adults aged 65 years and above in specific cities/provinces in mainland China. Then, we used a correlation analysis to explore the potential factors that influence influenza‐associated mortality, including influenza virus activity, demographics, economics, meteorology, and health services. Based on the results of the correlation analysis, we used generalized linear models to estimate the corresponding excess mortality stratified by province, accounting for the potential variables.

### 2.1 Systematic review

We followed the Preferred Reporting Items for Systematic Reviews and Meta‐Analyses (PRISMA) guidelines^19^ and searched five databases (PubMed, Web of Science, China National Knowledge Infrastructure [CNKI], Wanfang, and Chongqing VIP) to identify relevant articles published in English or Chinese between January 1, 2000, and September 17, 2022. Search terms included keywords about the influenza‐associated disease burden and geographic areas. The search strategy was developed and adapted for each database with a combination of keywords in MeSH, title/abstract, and all fields. Keywords included “influenza,” “burden,” “mortality,” and “China,” with many other closely related words and synonyms along with their Chinese translations (Table S1). The reference lists of all eligible articles were also searched.

Each article identified through the search strategy underwent a process of title, abstract, and full‐text screening based upon a set of inclusion and exclusion criteria (Table S2). Eligible articles were those reporting influenza‐associated excess mortality among older adults aged 65 years and above in specific cities/provinces in mainland China. Studies were excluded if (1) they did not report the target population or deaths from R&C or AC diseases; (2) they did not include seasonal influenza; (3) population‐based estimates of influenza‐associated excess mortality burden could not be derived; and (4) they were systematic reviews, meta‐analyses, conference proceedings, commentaries, editorials, or letters. Studies with a time frame combining both seasonal and pandemic influenza periods were included, but we excluded the data for the 2009 H1N1 pandemic.

We extracted publication information, methodological characteristics and outcome measurements from the included studies (Supporting Information p. 4; Table S3). To evaluate the quality of the included studies, we also developed a scoring system with five criteria, including publication, data reliability, regional representativeness, modeling method and result precision, with each score ranging from 0 to 1 point. Studies with scores of 3.5 or more, which were considered high‐quality studies, were included in the modeling process to ensure the accuracy of the estimates (Table S4). Literature screening, scoring, and data extraction were all carried out and cross‐checked by two researchers (K.D. and G.Z.), and the research team corrected any cases of inconformity (J.Y. and H.G.).

### 2.2 Parameters for correlation analysis and model fit

#### 2.2.1 Influenza virological data

To describe the influenza circulation intensity and pattern, annual influenza virological data (i.e., positive rate of influenza among all age groups by type and that among older adults aged 65 years and above) in 30 provinces (excluding Tibet due to limited sentinel hospitals) from 2005 to 2016 were extracted from the Influenza Weekly Report by the Chinese National Influenza Center and published literatures.^3^, ^20^, ^21^, ^22^, ^23^, ^24^ In addition, we systematically searched two Chinese peer‐reviewed databases (CNKI and Wanfang) to collect annual influenza virus‐positive rates by type in specific provinces before 2005 (Table S5).^25^, ^26^, ^27^, ^28^ However, the National Influenza Surveillance Network was not expanded until 2009, and positive rates of older adults aged 65 years and above were inaccurate due to the small sample size before 2009; thus, we used positive rates of all age groups to replace the positive rates of older adults aged 65 years and above before 2009 in the model fit.

#### 2.2.2 Other parameters

We obtained province‐stratified demographic data (number and proportion of rural residents) and economic data (gross domestic product per capita) from 2000 to 2016 from the China Statistical Yearbook.^9^ We also estimated the population density from 2001 to 2016 based on the population size in the corresponding year and available population density in 2000 from the China Statistical Yearbook and China Health Statistical Yearbook, respectively.^9^, ^29^

Meteorological data (ambient temperature, relative humidity) during 2000–2016 were obtained from the China Meteorological Data Sharing Service System.^30^ We used meteorological data from all monitoring stations within the same province to estimate temperature and relative humidity for the province as a whole and estimated absolute humidity based on ambient temperature and relative humidity.^31^

We extracted medical service data from the China Health Statistical Yearbook, including the numbers of hospital beds and healthcare workers per 1000 persons from 2000 to 2016, the number of outpatients from 2002 to 2016, and the number of inpatients from 2007 to 2016.^29^ Missing data in some years from 2000 to 2016 were supplemented by a linear model by province and year (Supporting Information p. 4).

### 2.3 Statistical analysis

To explore the potential impact of the aforementioned predictor variables on influenza‐associated excess R&C mortality, we conducted a correlation analysis between these predictor variables and excess R&C mortality. Then, based on the results of the correlation analysis, considering that influenza‐associated excess R&C mortality approximately follows a normal distribution,^31^, ^32^ we applied a generalized linear model method to estimate influenza‐associated excess R&C mortality, with a natural log link to avoid generating negative estimates of excess mortality,^33^ using the potential variables as the predictor variables and annual excess mortality rates as the outcome (Supporting Information p. 5). The final model was determined according to the extension of Akaike information criterion (AIC) (Supporting Information p. 6)^34^, ^35^ and applied to estimate annual average excess R&C mortality over 6 years from 2011 to 2016 for older adults aged 65 years and above in 30 provinces and nationwide. The annual average provincial and national excess mortality from 2011 to 2016 were estimated using the annual average variables over 6 years at the provincial and national levels, respectively. The 95% CI for influenza‐associated excess R&C mortality was obtained using the bootstrap method, whereby the model was simulated 2000 times. Likewise, excess AC mortality was estimated.

To explore the impact of data quality, different models and extremely low excess mortality on the results, we conducted the following sensitivity analyses: (1) using higher quality data (i.e., setting higher scoring criteria of no lower than 4) to fit the model to estimate influenza‐associated excess R&C and AC mortality; (2) applying a random forest approach to estimate the results; and (3) removing the extremely low excess R&C (<10 per 100,000) and AC (<20 per 100,000) mortality in specific cities/provinces which was obtained through aforementioned systematic review to fit the model to estimate the results. See the details in the Supporting Information pp. 6–7.

Internal and external comparisons were conducted to verify the accuracy of our estimates of influenza‐associated mortality. For the internal comparison, to compare the accuracy of different models, cross validation was conducted, which used statistical indicators such as the value of *R* squared ( $R^{2}$), root mean squared error (RMSE), and mean absolute error (MAE) (Supporting Information pp. 5–6). In addition, we obtained the original annual influenza‐associated mortality after 2010 in several provinces from aforementioned systematic review. We estimated the annual influenza‐associated excess mortality of the corresponding province and year in the main analysis and sensitivity analyses and then compared them with the original influenza‐associated mortality. The comparison was aimed to evaluate the accuracy of our estimates of influenza‐associated mortality. Furthermore, to externally validate our estimates, we compared the annual average influenza‐associated excess mortality among older adults aged 65 years and above after 2010 in two municipalities (Chongqing and Shanghai)^16^, ^17^, ^36^ with that of corresponding cities from 2011 to 2016 in the main analysis and sensitivity analyses. Finally, we compared the provincial‐level variation trend of our estimated influenza‐associated excess R&C and AC mortality with that of influenza‐associated excess respiratory mortality published previously.^3^ All statistical analyses were conducted using R version 4.2.1.

## 3 RESULTS

### 3.1 Literature review

**FIGURE 1:**
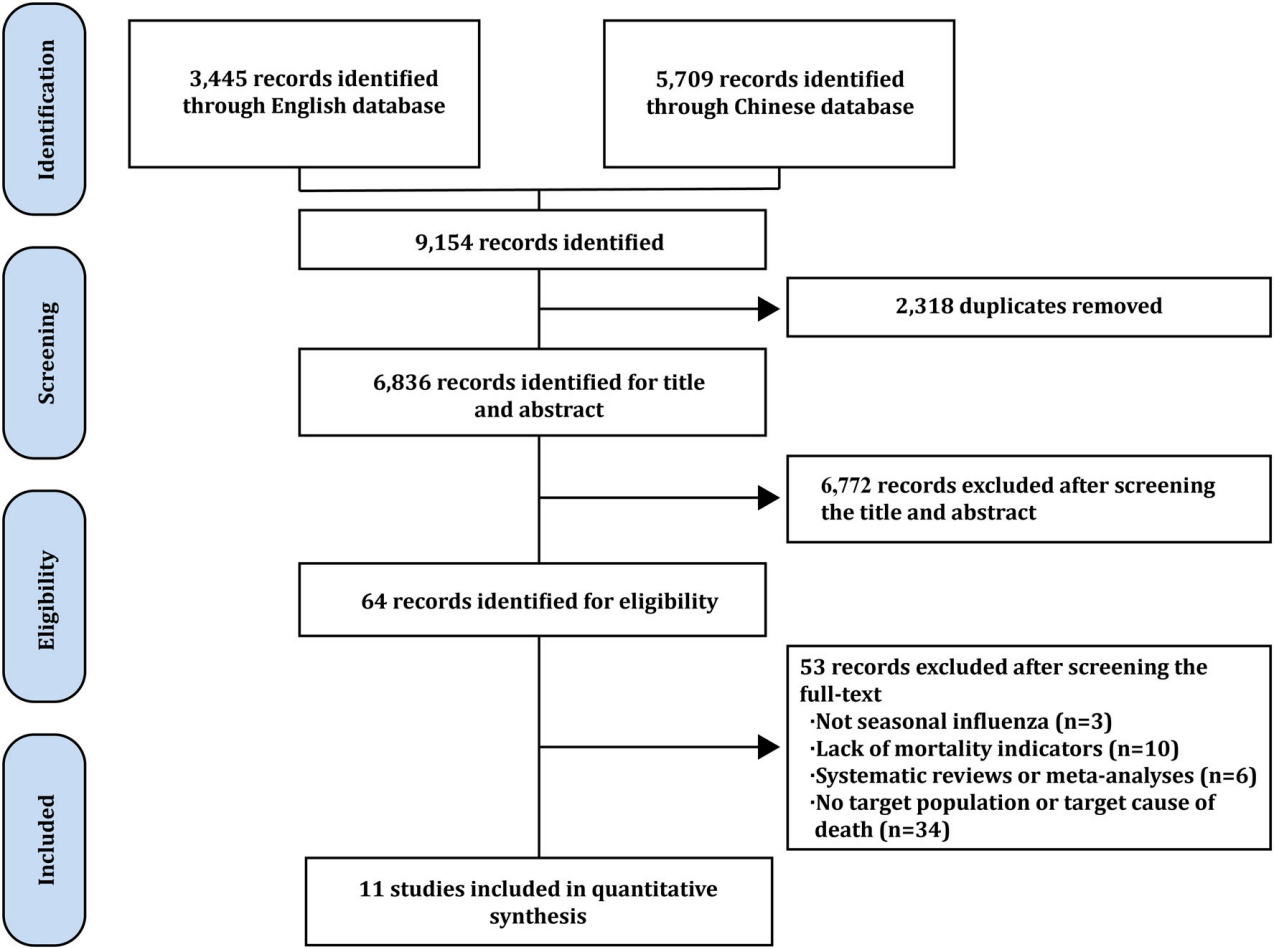
Flowchart of the systematic review process

We identified 9154 studies through the database search (Figure 1). After excluding duplicates, as well as screening the title and abstract, 64 studies were identified for eligibility. Finally, 11 (0.1%) were included in the quantitative synthesis after screening the full text (a detailed description was shown in Table S6). Studies were mainly conducted in economically developed areas in eastern China (three studies in Guangdong, two in Zhejiang, and each one in Beijing, Shandong, Shanghai and Liaoning), while limited studies were carried out in central (one in Heilongjiang) and western (one in Yunnan) China. Except for one study in Heilongjiang and one study in Zhejiang that reported only excess AC mortality, other studies reported both excess R&C and AC mortality. Time series data with a time granularity of 1 year or one influenza season were extracted for model analysis. All 11 studies scored 3.5 points or above and thus were included in the model analysis (Table S7).

Influenza‐associated excess R&C (45 province‐seasons) and AC (52 province‐seasons) mortality among older adults aged 65 years and above in eight provinces from 2000 to 2019 were included in the final model. The included data points were mainly before 2009, and six studies (R&C: 17/45, AC: 21/52) reported an excess mortality burden after 2009. Influenza‐associated excess R&C and AC mortality among older adults aged 65 years and above ranged from 5.2 in Liaoning to 378.8 in Yunnan per 100,000 persons and from 10.5 in Liaoning to 477.3 in Yunnan per 100,000 persons, respectively (Figure S1).

### 3.2 Estimating average influenza‐associated excess R&C and AC mortality among older adults aged 65 years and above by province and nationwide from 2011 to 2016

**FIGURE 2:**
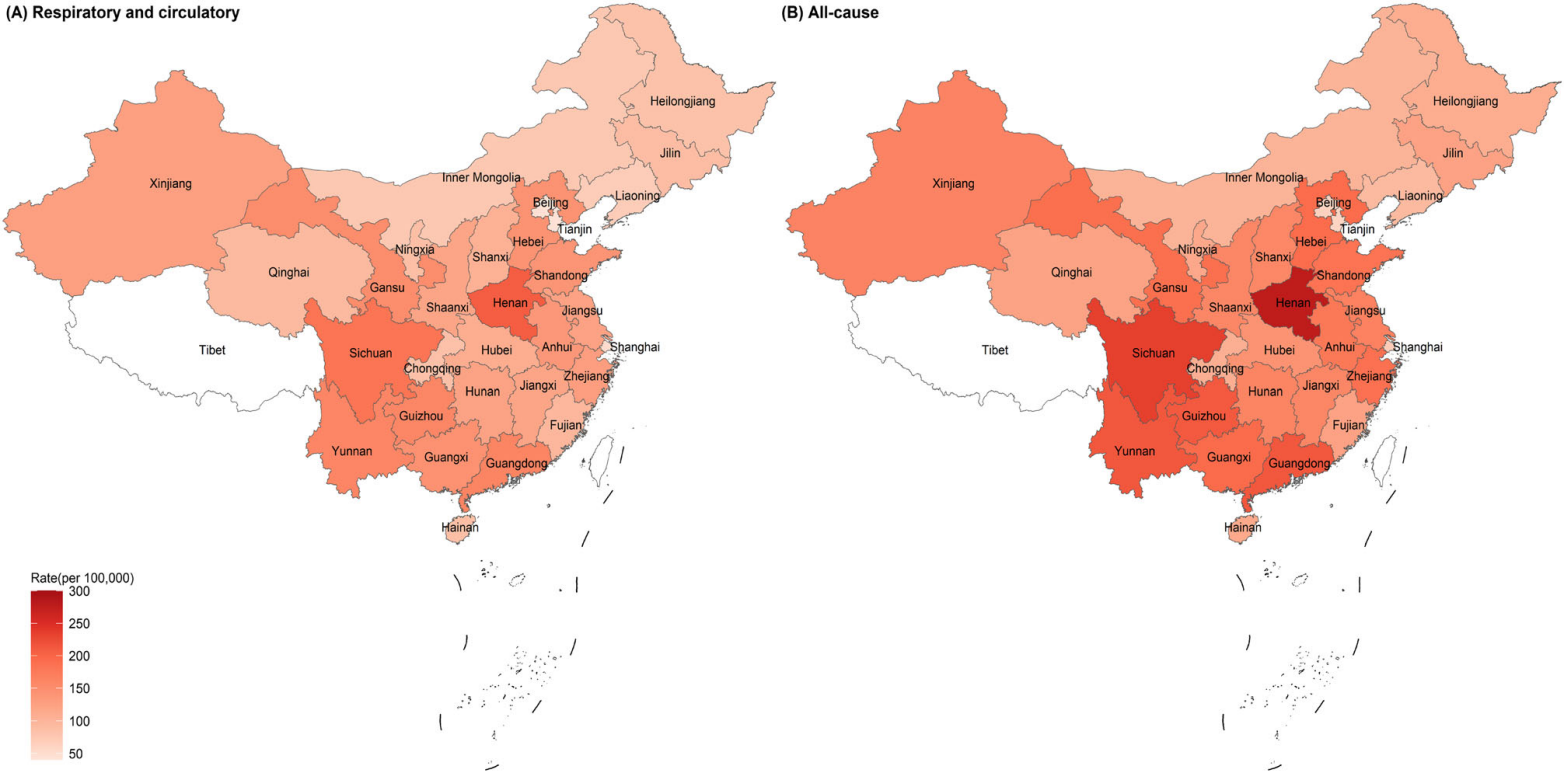
Estimated annual average influenza‐associated excess respiratory and circulatory and all‐cause mortality among older adults aged 65 years and above in 30 provinces in China, 2011–2016. Tibet was excluded due to a lack of virological data.

The generalized linear model showed a good fit and explained 61.6% and 64.0% of the variation in observed influenza‐associated excess R&C and AC mortality among older adults aged 65 years and above, respectively (Supporting Information p. 8; Tables S9 and S10). Overall, the annual national average influenza‐associated excess R&C mortality in China was estimated to be 111.8 (95% CI: 92.8–141.1) per 100,000 persons among older adults from 2011 to 2016 (Figure 2A). Large variations were observed among the 30 provinces, with the highest excess R&C mortality in Henan, Sichuan, and Guangdong provinces, ranging from 162.0 to 207.8 per 100,000 persons, and the lowest in Tianjin, Beijing, and Shanghai municipalities (Figure 2A).

Higher than the average influenza‐associated excess mortality rate attributable to R&C, the average influenza‐associated excess AC mortality among older adults from 2011 to 2016 was estimated to be 151.6 (95% CI: 127.6–179.3) per 100,000 persons at the national level and showed similar variation patterns among the provinces (Figure 2B).

### 3.3 Sensitivity analyses

**FIGURE 3:**
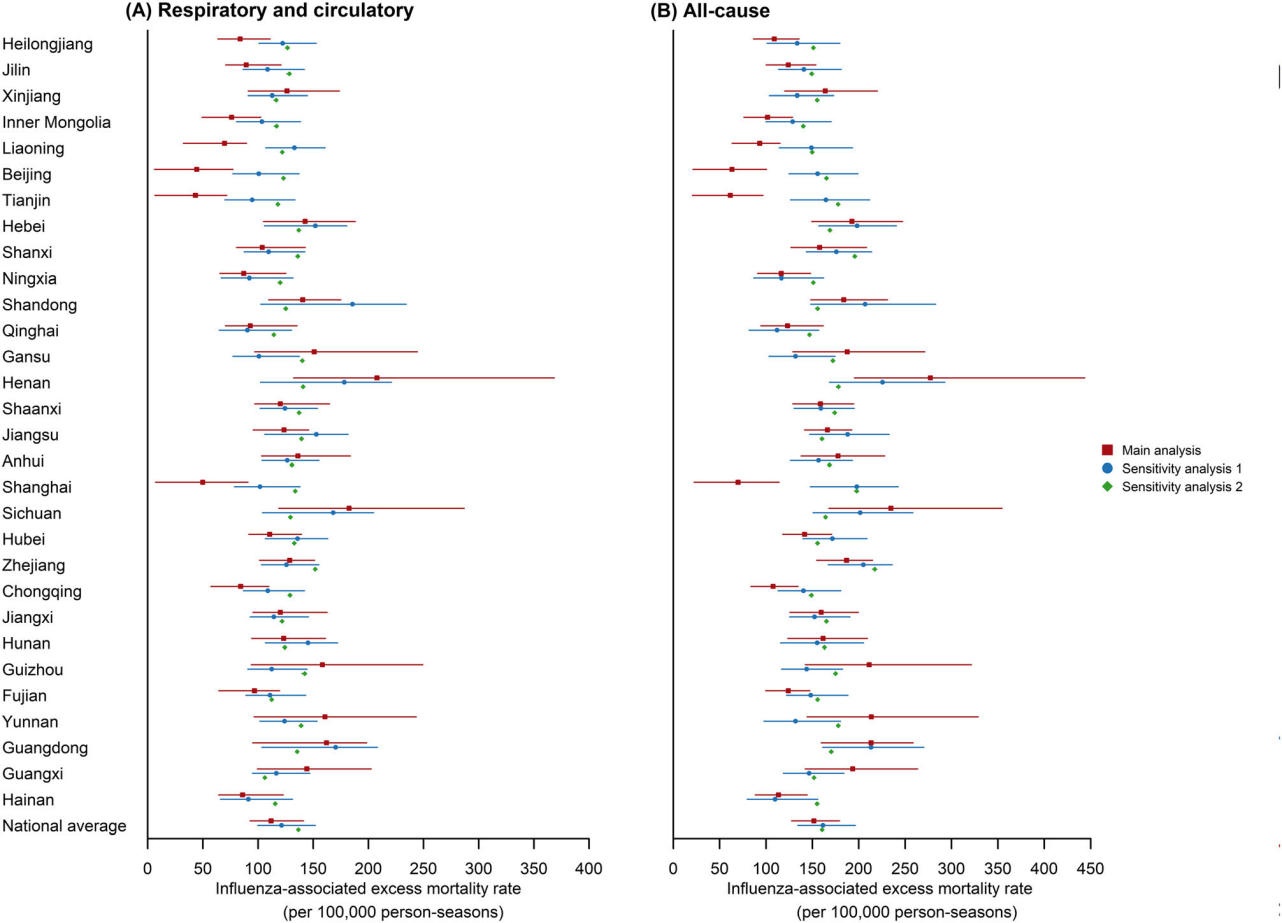
Main analysis and sensitivity analyses of annual average influenza‐associated excess respiratory and circulatory and all‐cause mortality among older adults aged 65 years and above in 30 provinces in China, 2011–2016. Sensitivity Analysis 1 (blue) indicates the estimated results obtained using higher quality data, and Sensitivity Analysis 2 (green) indicates the estimated results acquired using the random forest approach. Dots indicate the point estimates of excess mortality rates. Lines indicate the 95% CIs.

For the influenza‐associated excess R&C mortality among older adults aged 65 years and above, the sensitivity analyses using higher quality data, applying the random forest approach, and removing the extremely low excess mortality from the model fit produced similar results to the main analysis, with annual national average mortality estimated as 121.2 (95% CI: 99.7–151.8), 136.6 (95% CI: 135.0–137.4) and 114.9 (95% CI: 95.1–147.7) per 100,000 persons, respectively (Figures 3A and S2). Similar variations among provinces were also observed in Sensitivity Analysis 1 using higher quality data, with the highest excess mortality in Shandong, Henan, and Guangdong Provinces and the lowest in Qinghai, Hainan, and Ningxia Provinces (Figure 3A). In addition, there were similar spatial variations in the estimated results for most provinces in Sensitivity Analysis 2, conducted using the random forest approach, despite the fluctuations in some provinces. Moreover, the estimates and their 95% CI of influenza‐associated excess R&C mortality from the sensitivity analyses were included or overlapped with the 95% CI of the main analysis in most provinces (Sensitivity Analysis 1: 29/30; Sensitivity Analysis 2: 22/30), except Liaoning Province in Sensitivity Analysis 1 and Heilongjiang, Jilin, Inner Mongolia, Liaoning, Beijing, Tianjin, Shanghai, and Chongqing Provinces in Sensitivity Analysis 2 (Figure 3A).

For the influenza‐associated excess AC mortality among older adults aged 65 years and above, the sensitivity analyses, conducted using higher quality data, applying the random forest approach, and removing the extremely low excess mortality in model fit, produced similar results to the main analysis, with annual national average mortality estimated as 161.3 (95% CI: 134.2–196.2), 160.5 (95% CI: 158.5–161.2), and 151.6 (95% CI: 127.6–179.3) per 100,000 persons, respectively (Figures 3B and S2). The spatial variations among provinces in the sensitivity analyses were similar to those in the main analysis. Furthermore, the estimates and their 95% CI of influenza‐associated excess AC mortality from the sensitivity analyses were included or overlapped with the 95% CI of the main analysis in most provinces (Sensitivity Analysis 1: 27/30; Sensitivity Analysis 2: 18/30) (Figure 3B).

### 3.4 Internal and external comparisons of influenza‐associated excess mortality among older adults aged 65 years and above between the estimates and published results

The cross validation showed that the overall RMSE of the main analysis was slightly less than that of the sensitivity analyses, and the MAE was similar between the main analysis and the sensitivity analyses (Tables S9 and S10). The internal comparison demonstrated that the majority of original annual influenza excess R&C and AC mortality in Beijing, Guangdong, Yunnan, and Zhejiang Provinces, which were obtained in previous publications,^18^, ^37^, ^38^, ^39^, ^40^ were similar to our corresponding estimates (Figures S3 and S4). The external comparison showed consistency in average influenza excess R&C and AC mortality in multiple seasons between our estimates and those obtained in previous publications in Chongqing and Shanghai (Figures S5 and S6).^16^, ^17^, ^36^ Furthermore, we also found that the large spatial variations in influenza‐associated excess R&C and AC mortality among the provinces followed the same trend as the variations observed in influenza‐associated excess respiratory mortality among provinces in China from a previous study (Figure S7).^3^

## 4 DISCUSSION

This study estimated the excess mortality burden of seasonal influenza attributable to R&C and AC diseases among older adults aged 65 years and above at the provincial and national levels in China based on robust vital statistics and mortality data obtained from a systematic review. It was found that the mortality burden of seasonal influenza was substantial among older adults, with the annual national average excess R&C and AC mortality from 2011 to 2016 estimated as 111.8 (95% CI: 92.8–141.1) and 151.6 (95% CI: 127.6–179.3) per 100,000 persons, respectively.

Our excess mortality estimates attributable to R&C and AC diseases among older adults is consistent with the influenza‐associated excess mortality burden estimated before the 2009 pandemic in China in a previous study, despite using a different modeling approach.^10^ Our estimates have some differences from those in other countries/areas (Table S11). Our estimate for R&C was higher than that reported in Colorado, a state in western America^41^; Shanghai, a subtropical city in eastern China^17^; and Yancheng, a subtropical city in eastern China,^42^ but lower than that reported in India^43^ and Shenzhen, a tropical city in China.^18^ Our estimate for AC diseases was higher than that reported in Europe,^44^ Greece,^45^ Denmark, and Hong Kong.^46^, ^47^ Possible explanations for the differences might be the regional variations in socioeconomic and demographic factors,^10^, ^48^, ^49^ medical resource utilization, diagnostic criteria for the recorded cause of death,^50^, ^51^, ^52^ different models used,^11^, ^53^ and different study periods.^6^, ^10^, ^52^, ^54^ Furthermore, successful implementation of seasonal influenza vaccination might also have played an important role. The influenza vaccination subsidy policies were quite different in different countries or regions, with widespread influenza vaccination programs for older adults in many high‐income countries and limited programs in low‐ and middle‐income countries.^55^

This study reveals large spatial variations in influenza‐associated excess R&C and AC mortality among the provinces in China. The higher mortality burden of influenza among older adults in Henan and Sichuan Provinces might be attributable to their higher proportion of rural residents, which is a key parameter in our model fit. Yu *et al* have also demonstrated that the influenza‐associated excess R&C and AC mortality burdens in rural areas are significantly higher than those in urban areas.^10^ In Guangdong, the high mortality burden might be attributable to its high outpatient burden and absolute humidity. There was a significant positive correlation between the intensity of influenza virus activity and outpatient visits. The high outpatient burden might indicate high influenza virus activity in Guangdong Province, which causes a high mortality burden.^56^, ^57^ In addition, the high absolute humidity might exacerbate the mortality burden.^58^, ^59^, ^60^

Likewise, the lowest excess R&C and AC mortality burden in Tianjin, Beijing, and Shanghai municipalities would be related to their low proportion of rural residents. In addition, higher socioeconomic development, better medical resources, and higher influenza vaccine coverage might contribute to the low mortality. For instance, the government has provided annual free seasonal influenza vaccination for older registered adults aged 60 years and above in Beijing since 2007, increasing vaccine coverage to 44% in 2010–2011,^61^ which is much higher than the average coverage across China (approximately 3.8%).^8^

Compared with other age groups, older adults aged 65 years and above have the highest influenza‐associated excess R&C (111.8 in older adults vs. 0.7–3.3 in younger age groups per 100,000 persons) and AC mortality burden (151.6 in older adults vs. 2.5–6.8 in younger age groups per 100,000 persons).^5^, ^6^, ^10^, ^16^, ^17^, ^36^ Older adults are considered among the high‐risk groups for seasonal influenza, because they are prone to severe complications, illnesses, and even deaths after influenza virus infection.^62^, ^63^ Seasonal influenza vaccination is the most effective intervention for protecting against influenza virus infection in outpatients and inpatients and reducing mortality.^64^ China has extremely low vaccination coverage (3.8%) among older adults, much lower than that in other high‐income countries. It is necessary to promote influenza vaccination for older adults to reduce influenza‐associated mortality in mainland China.

To increase influenza vaccine coverage, an increasing number of local governments have enabled older adults to receive influenza vaccination paid wholly or partially by the government or Basic Social Medical Insurance, but these are mostly concentrated in economically developed regions with rich medical resources and a low mortality burden (i.e., Beijing, Tianjin).^7^ Underresourced provinces with high influenza‐associated mortality burdens lack relevant measures. Therefore, there is an urgent need to include influenza vaccination for older adults in the National Immunization Program to allow more individuals to enjoy medical equity, especially for those in provinces with a high mortality burden.

This study had some limitations. First, through a systematic review, we collected mortality data from eight provinces (R&C: 45 province‐seasons, AC: 52 province‐seasons) in mainland China from different years to construct a generalized linear model to estimate average influenza‐associated excess R&C and AC mortality among older adults aged 65 years and above by province and nationwide in China. The limited mortality data varied across models and might have influenced the certainty of the generalized linear model by extrapolation, further leading to an underestimate of the national influenza‐associated mortality burden. Therefore, continued national monitoring of influenza virology and mortality surveillance are needed to further analyze the influenza‐associated mortality burden among older adults in future studies. Second, because only limited virological data were available up to 2016, we only estimated the influenza‐associated excess mortality from 2011 to 2016. Third, although the internal and external comparisons demonstrated that our estimates of influenza‐associated excess R&C and AC mortality were reliable, inconsistency between the main analysis and sensitivity analyses was observed in several provinces (such as Liaoning and Beijing). This might suggest that the variations in the estimates of these provinces were mainly affected by the optimal variables chosen by the model in the main analysis and sensitivity analyses, especially in Sensitivity Analysis 1, which had fewer data points. Therefore, influenza‐associated mortality merits more studies in the future. Fourth, after the 2009 influenza pandemic, A/H1N1pdm09 replaced the preexisting seasonal influenza A virus subtype H1N1 (seasonal A/H1N1), circulating as a seasonal influenza virus.^65^, ^66^ Whether A/H1N1pdm09 replacement has had an impact on influenza‐associated mortality has not been fully addressed. The influenza‐associated excess AC and the influenza‐associated pneumonia and influenza mortality of the post pandemic were lower than that of the pre pandemic in England and Wales.^67^ However, the influenza‐associated respiratory, cardiovascular, and AC mortality rates during the post pandemic were similar to those of the pre pandemic in Mexico.^49^ And a systematic review demonstrated no difference in the influenza‐associated excess mortality between the pre pandemic and post pandemic in China.^68^ The circulation intensity of influenza virus by type (influenza A and influenza B) was used as a covariate in the model. However, the circulation intensity stratified by influenza A subtype (i.e., seasonal A/H1N1, A/H1N1pdm09, and H3N2) was not used in the model due to data unavailability. Accordingly, the potential impact of A/H1N1pdm09 replacement was only partially accounted for in this study. Additional studies on this topic are of interest. Fifth, the potential time trend might mainly influence the mortality of the population and the intensity of influenza virus activity in our study. The overall mortality rate of the whole population was relatively stable, with little change, ranging from 7.04 to 7.14 per 1000 persons.^9^ In addition, influenza‐associated mortality is strongly correlated with the intensity of influenza virus activity, which can reflect changes in time trends of influenza‐associated mortality burden. Although the intensity of influenza virus activity is seasonal in China,^69^ we used an annual indicator of influenza activity intensity in our model without considering its variation within the year. Therefore, we did not consider any other time trend effect in the model. Finally, pathogens such as respiratory syncytial virus, which was suggested in previous studies to be associated with increased mortality in adults,^54,70^ were not included in our model.

## 5 CONCLUSION

This study demonstrated a substantial influenza‐associated mortality burden among older adults aged 65 years and above in 30 provinces in China from 2011 to 2016. Differences in demographic, meteorological, socioeconomic, and medical resource factors could explain spatial variations in influenza‐associated mortality burden for older adults aged 65 years and above among provinces. Our estimates of the influenza‐associated excess R&C and AC mortality provided valuable information to support policy‐makers in including influenza vaccination for older adults in the National Immunization Program to promote medical equity in China.

## CONFLICTS OF INTEREST

H.Y. received research funding from Sanofi Pasteur, GlaxoSmithKline, Yichang HEC Changjiang Pharmaceutical Company, Shanghai Roche Pharmaceutical Company, and SINOVAC Biotech Ltd. All the other authors have no competing interests.

## AUTHOR CONTRIBUTIONS

**Kaige Dong:** Data curation; formal analysis; software; validation; visualization; writing‐original draft; writing‐review and editing. **Hui Gong:** Data curation; software; validation; visualization; writing‐original draft. **Guangjie Zhong:** Data curation; validation; visualization; writing‐original draft; writing‐review and editing. **Xiaowei Deng:** Methodology; software. **Yuyang Tian:** Formal analysis; visualization; writing‐original draft. **Minghan Wang:** Data curation. **Hongjie Yu:** Conceptualization; funding acquisition; supervision. **Juan Yang:** Conceptualization; funding acquisition; supervision; writing‐original draft; writing‐review and editing.

## Supporting information


**Table S1.** Literature search strategy and results, by database.
**Table S2.** Inclusion and exclusion criteria of the studies.
**Table S3.** Data category of extraction from the included studies.
**Table S4.** Literature scoring criteria for the included studies.
**Table S5.** Literature search strategy and the results of influenza virus‐positive rates by type in specific provinces before 2005, by database.
**Table S6.** Basic information on the included studies.
**Table S7.** Literature scoring results of the included studies.
**Table S8.** Selected covariates for the next modeling process.
**Table S9.** The results of cross validation of the main analysis and sensitivity analyses in influenza‐associated excess respiratory and circulatory among older adults aged 65 years and above.
**Table S10.** The results of cross validation of the main analysis and sensitivity analyses in influenza‐associated excess all‐cause among older adults aged 65 years and above.
**Table S11.** Comparison of estimates of annual influenza‐associated excess respiratory and circulatory and all‐cause mortality (per 100,000) among older adults aged 65 years and above after the 2009 pandemic.
**Figure S1.** Influenza‐associated excess respiratory and circulatory (R&C) and all‐cause (AC) mortality among older adults aged 65 years and above by province and by year from the included studies.
**Figure S2.** Estimated annual average influenza‐associated excess respiratory and circulatory and all‐cause mortality among older adults aged 65 years and above in 30 provinces in China, 2011‐2016.
**Figure S3.** Internal comparison of influenza‐associated excess respiratory and circulatory (R&C) mortality among older adults aged 65 years and above between the estimates and published results in available provinces.
**Figure S4.** Internal comparison of influenza‐associated excess all‐cause (AC) mortality among older adults aged 65 years and above between the estimates and published results in available provinces.
**Figure S5.** External comparison of influenza‐associated excess respiratory and circulatory (R&C) mortality among older adults aged 65 years and above between the estimates and published results in available cities.
**Figure S6.** External comparison of influenza‐associated excess all‐cause (AC) mortality among older adults aged 65 years and above between the estimates and published results in available cities.
**Figure S7.** Comparison of the provincial‐level variation trend between our estimates of influenza‐associated excess respiratory and circulatory (R&C) and all‐cause (AC) mortality in the main analysis and the estimates of influenza‐associated excess respiratory (R) mortality published previously

## Data Availability

The data that support the findings of this study are openly available on GitHub at https://github.com/lvyihongning/Influenza-associated-mortality-data.
