# Supplementary material for: Estimating mortality associated with seasonal influenza among adults aged 65 years and above in China from 2011 to 2016: A systematic review and model analysis
Source: Influenza Other Respir Viruses. 2022 Nov 17;17(1):e13067. doi: 10.1111/irv.13067 (PMC9835403; doi:10.1111/irv.13067)
Supplement: Supplementary file 1 — Table S1. Literature search strategy and results, by database. Table S2. Inclusion and exclusion criteria of the studies. Table S3. Data category of extraction from the included studies. Table S4. Literature scoring criteria for the included studies. Table S5. Literature search strategy and the results of influenza virus‐positive rates by type in specific provinces before 2005, by database. Table S6. Basic information on the included studies. Table S7. Literature scoring results of the included studies. Table S8. Selected covariates for the next modeling process. Table S9. The results of cross validation of the main analysis and sensitivity analyses in influenza‐associated excess respiratory and circulatory among older adults aged 65 years and above. Table S10. The results of cross validation of the main analysis and sensitivity analyses in influenza‐associated excess all‐cause among older adults aged 65 years and above. Table S11. Comparison of estimates of annual influenza‐associated excess respiratory and circulatory and all‐cause mortality (per 100,000) among older adults aged 65 years and above after the 2009 pandemic. Figure S1. Influenza‐associated excess respiratory and circulatory (R&C) and all‐cause (AC) mortality among older adults aged 65 years and above by province and by year from the included studies. Figure S2. Estimated annual average influenza‐associated excess respiratory and circulatory and all‐cause mortality among older adults aged 65 years and above in 30 provinces in China, 2011‐2016. Figure S3. Internal comparison of influenza‐associated excess respiratory and circulatory (R&C) mortality among older adults aged 65 years and above between the estimates and published results in available provinces. Figure S4. Internal comparison of influenza‐associated excess all‐cause (AC) mortality among older adults aged 65 years and above between the estimates and published results in available provinces. Figure S5. External comparison of influenza‐associa [file IRV-17-e13067-s001.docx]

**Additional file 1**

**Estimating mortality associated with seasonal influenza among adults aged 65 years and above in China from 2011-2016: A systematic review and model analysis**

Kaige Dong^1,2^, Hui Gong^1,2^, Guangjie Zhong^1,2^, Xiaowei Deng^1,2^, Yuyang Tian^1,2^, Minghan Wang^1,2^, Hongjie Yu^1,2†^, Juan Yang^1,2*†^

†These authors jointly supervised this work.

*Correspondence: Juan Yang, yangjuan@fudan.edu.cn

Affiliations:

1. Shanghai Institute of Infectious Disease and Biosecurity, School of Public Health, Fudan University, Shanghai, China
2. School of Public Health, Fudan University, Key Laboratory of Public Health Safety, Ministry of Education, Shanghai, China

**Content**

[Methods 4](#_Toc119011234)

[1. Data extraction 4](#_Toc119011235)

[2. Statistical methods 4](#_Toc119011236)

[2.1 Estimate for missing covariates 4](#_Toc119011237)

[2.2 Correlation between influenza-associated excess mortality rates and each covariate 4](#_Toc119011238)

[2.3 Statistical model 5](#_Toc119011239)

[2.4 Selection of coefficients 6](#_Toc119011240)

[2.5 Sensitivity analyses 6](#_Toc119011241)

[Results 8](#_Toc119011242)

[1. Estimating average influenza-associated excess R&C mortality among older adults aged 65 years and above by province and nationwide from 2011-2016. 8](#_Toc119011243)

[2. Estimating average influenza-associated excess AC mortality among older adults aged 65 years and above by province and nationwide from 2011-2016. 8](#_Toc119011244)

[Supplementary Tables 9](#_Toc119011245)

[Table S1. Literature search strategy and results, by database. 9](#_Toc119011246)

[Table S2. Inclusion and exclusion criteria of the studies. 11](#_Toc119011247)

[Table S3. Data category of extraction from the included studies. 12](#_Toc119011248)

[Table S4. Literature scoring criteria for the included studies. 13](#_Toc119011249)

[Table S5. Literature search strategy and the results of influenza virus-positive rates by type in specific provinces before 2005, by database. 15](#_Toc119011250)

[Table S6. Basic information on the included studies. 17](#_Toc119011251)

[Table S7. Literature scoring results of the included studies. 19](#_Toc119011252)

[Table S8. Selected covariates for the next modeling process. 29](#_Toc119011253)

[Table S9. The results of cross validation of the main analysis and sensitivity analyses in influenza-associated excess respiratory and circulatory among older adults aged 65 years and above. 30](#_Toc119011254)

[Table S10. The results of cross validation of the main analysis and sensitivity analyses in influenza-associated excess all-cause among older adults aged 65 years and above. 31](#_Toc119011255)

[Table S11. Comparison of estimates of annual influenza-associated excess respiratory and circulatory and all-cause mortality (per 100,000) among older adults aged 65 years and above after the 2009 pandemic. 32](#_Toc119011256)

[Supplementary Figures 33](#_Toc119011257)

[Figure S1. Influenza-associated excess respiratory and circulatory (R&C) and all-cause (AC) mortality among older adults aged 65 years and above by province and by year from the included studies. 33](#_Toc119011258)

[Figure S2. Estimated annual average influenza-associated excess respiratory and circulatory and all-cause mortality among older adults aged 65 years and above in 30 provinces in China, 2011-2016. 34](#_Toc119011259)

[Figure S3. Internal comparison of influenza-associated excess respiratory and circulatory (R&C) mortality among older adults aged 65 years and above between the estimates and published results in available provinces. 35](#_Toc119011260)

[Figure S4. Internal comparison of influenza-associated excess all-cause (AC) mortality among older adults aged 65 years and above between the estimates and published results in available provinces. 36](#_Toc119011261)

[Figure S5. External comparison of influenza-associated excess respiratory and circulatory (R&C) mortality among older adults aged 65 years and above between the estimates and published results in available cities. 37](#_Toc119011262)

[Figure S6. External comparison of influenza-associated excess all-cause (AC) mortality among older adults aged 65 years and above between the estimates and published results in available cities. 38](#_Toc119011263)

[Figure S7. Comparison of the provincial-level variation trend between our estimates of influenza-associated excess respiratory and circulatory (R&C) and all-cause (AC) mortality in the main analysis and the estimates of influenza-associated excess respiratory (R) mortality published previously 39](#_Toc119011264)

[References 40](#_Toc119011265)

# Methods

1. Data extraction

An extraction process was subsequently conducted to transform data of certain classes into a predefined form (Table S3). For each included study, the following data were extracted: general characteristics of the studies, methodological characteristics, and primary outcome measurements. The primary outcomes included annual average influenza-associated mortality rates among older adults aged 65 years and above and cause of death. The influenza-associated mortality burden estimates were extracted directly by year. For studies reporting only the number of excess deaths, we calculated the excess rates by dividing excess deaths by the population size. Statistical models used to estimate influenza mortality burden in these studies were classified as the Serfling model, rate difference model, distributed lag non-linear model and regression model (including Poisson model and negative binomial model).

1. Statistical methods

2.1 Estimate for missing covariates

After collecting and collating the current covariates and dependent variable data, we found that some covariates were missing in some years, including the proportion of rural population (Guangdong 2004, Shandong 2001-2004, Liaoning 2003-2004, Shanghai 2001-2004), the number of outpatients (Shandong 2001, Shanghai 2000-2001), and the number of inpatients (Heilongjiang 2005-2006, Guangdong 2004-2006, Shandong 2001-2006, Liaoning 2003-2006, Shanghai 2000-2006). We established a simple linear regression model for different covariates and provinces and estimated the missing covariate parameters to improve the model database used for subsequent analysis.

2.2 Correlation between influenza-associated excess mortality rates and each covariate

Before fitting the generalized linear model, we first analyzed the correlation between influenza-associated excess mortality rates and each covariate with the Pearson correlation coefficient. We set two criteria for the first screening of covariates that would be used in the modeling processes.

1) P value < 0.05;

2) The correlation is consistent with mainstream scientific research conclusions.

Hence, we obtained the appropriate covariates for subsequent model analysis (Table S8).

2.3 Statistical model

In this study, we used a generalized linear model with a Gaussian family to fit the excess mortality rates by province. We assumed $\mu_{t}$ as the influenza-associated excess R&C and AC mortality at year $t$. Assuming that $\frac{M_{t}}{N_{t}}*{10}^{6}\sim gaussian(\mu_{t}, \sigma^{2})$, then our model was:

$$E \left( \mu_{t} \right)= g^{-1}{[\beta}_{0}+\beta_{1}x_{1}+\beta_{2}x_{2}+ \ldots+ \beta_{n}x_{n}]$$

The number of influenza-associated excess deaths at a year for older adults aged 65 years and above in the province is represented as $M_{t}$, $N_{t}$ is an offset that accounts for the elderly population size of the province in that year, and $\beta_{j}$ represents a linear effect of different variables that accounts for changes in mortality. $g^{-1}$ is the natural log link function that is used to avoid generating negative estimates of excess mortality.^1^ The variable $x_{n}$ corresponds to factors such as the proportion of rural residents, gross domestic product per capita (GDP), the number of internal and pediatric consultations, and average temperature associated with excess mortality rates. We used the cross validation method to estimate the performance of our model and flag problems such as overfitting or selection bias in practice. The accuracy of the model can be evaluated by the value of R squared ($R^{2}$), root mean squared error (RMSE), and mean absolute error (MAE). Lower RMSE and MAE values indicate better performance of the model.

$$R^{2}=1- \frac{\sum_{i} {(\hat{y}_{i}-\bar{y}_{i})}^{2}}{\sum_{i} {(y_{i}-\bar{y}_{i})}^{2}}$$

$$RMSE= \sqrt{\frac{1}{n} \sum_{1}^{n} {(y_{i}-\bar{y}_{i})}^{2}}$$

$$MAE= \frac{1}{n}{|(y}_{i}-\hat{y}_{i})|$$

2.4 Selection of coefficients

The candidate independent variables for the generalized linear model were chosen based on their potential impact on the observed influenza-associated mortality burden in each province, either changing influenza transmission in the population or affecting access to health care services. Based on the results of the screening of covariates, we artificially specified that only one variable in each category was put into the generalized linear model to avoid the covariance problem. The final model was determined by the extension of the Akaike Information Criterion (AICc).^2,3^ We used the final model with the minimum AICc value to estimate the annual average influenza-associated excess R&C and AC mortality in 30 provinces and nationwide from 2011-2016.

$$AIC=2k-2\ln\left( L \right)$$

$$AICc=AIC+ \frac{2k(k+1)}{n-k-1}$$

where *k* denotes the number of covariates, *L* denotes the likelihood function and *n* denotes the size of the sample.

2.5 Sensitivity analyses

We conducted a sensitivity analysis to examine whether the quality of the included studies would affect the estimates of influenza-associated excess mortality burden by province. According to the results of the literature scoring, we redefined the higher-quality studies with a higher score of 4 or more and then included them in the modeling process. Furthermore, to identify the accuracy of the generalized linear model, we applied the random forest approach (R package randomForest^4^) to estimate the average influenza-associated excess mortality among older adults aged 65 years and above by province from 2011-2016. As with the generalized linear model, the related variables from the results of the correlation analysis were trained, and their importance in the random forest model was calculated. The performance of the random forest model was evaluated and optimized for the smallest error estimate via 10-fold cross-validation and the “out-of-bag” error. In addition, to reduce the effect of extremely low estimates on our results, we removed the extremely low excess R&C (<10 per 100,000) and AC (<20 per 100,000) mortality to fit the model to estimate the influenza-associated mortality burden.

# Results

1. Estimating average influenza-associated excess R&C mortality among older adults aged 65 years and above by province and nationwide from 2011-2016.

Based on the results of the correlation analysis, we added the related variables (Table S8) to the model and chose the final model according to the minimum AICc value. The final model was described as follows:

$$\log\left( \mu_{t} \right)= \beta_{0}+\beta_{1}Dem\_rural.prop+\beta_{2}Ser\_outpatient+\beta_{3}Rate\_B + log(N_{t})$$

$$Y_{t} \sim gaussian(\mu_{t}, \sigma^{2})$$

1. Estimating average influenza-associated excess AC mortality among older adults aged 65 years and above by province and nationwide from 2011-2016.

Based on the results of the correlation analysis, we added the related variables (Table S8) to the model and chose the final model according to the minimum AICc value. The final model was described as follows:

$$\log\left( \mu_{t} \right)= \beta_{0}+\beta_{1}Dem\_rural.prop+ \beta_{2}Ser\_outpatient+ \beta_{3}Rate.all.65+log(N_{t})$$

$$Y_{t} \sim gaussian(\mu_{t}, \sigma^{2})$$

# Supplementary Tables

## Table S1. Literature search strategy and results, by database.

| **Database** | **Searching strategy** | **Results** |
| --- | --- | --- |
| PubMed | (((((influenza OR flu) AND (mortality OR death OR fatality OR burden))) NOT (avian[Title/Abstract] OR swine[Title/Abstract] OR zoonotic[Title/Abstract] OR h5n1[Title/Abstract] OR h5n2[Title/Abstract] OR h7n1[Title/Abstract] OR h7n2[Title/Abstract] OR h7n3[Title/Abstract] OR h7n7[Title/Abstract] OR h7n9[Title/Abstract] OR h9n2[Title/Abstract] OR h10n7[Title/Abstract] OR h10n8[Title/Abstract]))) AND (China OR Chinese) AND ("2000/01/01"[Date - Publication]: "2022/09/17"[Date - Publication]) AND English[Language]) | 1456 |
| Web of Science | ALL=(influenza OR flu) AND ALL=(mortality OR death OR fatality OR burden) NOT TS=(avian OR swine OR zoonotic OR h5n1 OR h5n2 OR h7n1 OR h7n2 OR h7n3 OR h7n7 OR h7n9 OR h9n2 OR h10n7 OR h10n8) AND ALL=China OR Chinese) AND DOP=(2000-01-01/2022-09-17) AND LA = English | 1989 |
| CNKI | (TKA=(流感+流行性感冒)) AND (SU= (超额死亡+ 死亡)) NOT TKA=(禽流感+嗜血杆菌+猪流感+马流感+犬流感) | 867 |
| Wanfang | 题名:(流感 or 流行性感冒) not 全部:(禽流感 or 嗜血杆菌 or 猪流感 or 马流感 or 犬流感) and 主题:(超额死亡 or 死亡) | 2327 |
| Chongqing VIP | M= (流感 OR 流行性感冒) AND U = (超额死亡 OR 死亡) NOT M = (禽流感 OR 嗜血杆菌 OR 猪流感 OR 马流感 OR 犬流感) | 2515 |

## Table S2. Inclusion and exclusion criteria of the studies.

| Inclusion criteria | 1. Studies that reported influenza-associated excess mortality among adults aged 65 years and above in specific cities/provinces in mainland China. |
| --- | --- |
| Exclusion criteria | 1. Studies that did not report target population or deaths from respiratory and cardiovascular or all-cause diseases 2. Studies that did not include seasonal influenza 3. Population-based estimates of influenza-associated excess mortality burden could not be derived 4. Studies that were systematic reviews, meta-analyses, conference proceedings, commentaries, editorials, and letters 5. Studies that were not periodical literature 6. Studies that were not full-text 7. Studies that were lacking influenza indicators 8. Studies that had unclear data sources |

## Table S3. Data category of extraction from the included studies.

| **No.** | **Category** | **Content** |
| --- | --- | --- |
| 1 | General information | First author, title, journal, publication year, language. |
| 2 | Study characteristics | Study method, data source, study period (start date, end date), year of each season, region (city, province), age of target population, limitation. |
| 3 | Outcome measurements | Cause of deaths, influenza-associated excess deaths or excess mortality per 100,000 persons in target groups (by influenza type/subtype if available). |

## Table S4. Literature scoring criteria for the included studies.

| **Item no.** | **Total Points** | **Criteria** | **Score** | **Implication** |
| --- | --- | --- | --- | --- |
| 1 | 1 | Publication:  The research was published in Chinese or foreign core journals. | 1 | Yes. |
|  |  |  | 0.5 | The research was published in doctoral and master’s dissertations. |
|  |  |  | 0 | Others. |
| 2 | 1 | Data reliability:  Data sources, collection and processing methods for influenza virus activity, mortality data, or other covariates needed for modeling were clearly indicated. | 1 | Yes. |
|  |  |  | 0.5 | Data sources, collection, and processing methods were generally described, but it is not clear which agencies, websites, collection, or statistical methods were involved. |
|  |  |  | 0 | No description of data sources and processing methods. |
| 3 | 1 | Regional representativeness:  The data source and time scale (≥3 seasons) used in the study allowed the results to be representative of the population in the intended area. | 1 | Yes. The results can be well representative of the provincial or prefecture-level population. |
|  |  |  | 0.5 | The evidence is not sufficient, or justification has been provided in terms of data representativeness. |
|  |  |  | 0 | No, and no justification has been provided. |
| 4 | 1 | Modeling method:  The model considered influenza activity (test positive rate, ILI% or other influenza-related proxy) and other relevant variables (meteorological, economic, etc.), or adopted functions to reflect cyclical patterns, long-term trends in deaths. | 1 | Yes, e.g., Negative binomial model, Serfling regression model, Poisson regression model, etc. |
|  |  |  | 0.5 | Only influenza virus activity variables were considered in the model, e.g., Rate difference model, etc. |
|  |  |  | 0 | No statistical models were used in the estimates of mortality. |
| 5 | 1 | Result precision:  Point estimates, confidence interval and its estimation methods for excess mortality in populations aged 65 years and above were directly provided. | 1 | The results have been adequately described. |
|  |  |  | 0.5 | The results have been described but not adequately, e.g., requiring conversion or lacking confidence intervals. |
|  |  |  | 0 | The results for the elderly have not been described. |

## Table S5. Literature search strategy and the results of influenza virus-positive rates by type in specific provinces before 2005, by database.

| **Database** | **Searching strategy** | **Results** |
| --- | --- | --- |
| CNKI | ((SU%=(流感+流行性感冒+甲流+乙流)NOT SU%=(禽流感+猪流感+流感嗜血杆菌))AND SU%=(阳性率+病毒学+实验室诊断+实验室确诊)AND FT=(黑龙江+广东+山东+辽宁+上海+哈尔滨+齐齐哈尔+鸡西+鹤岗+双鸭山+大庆+伊春+佳木斯+七台河+牡丹江+黑河+绥化+大兴安岭+广州+韶关+深圳+珠海+汕头+佛山+江门+湛江+茂名+肇庆+惠州+梅州+汕尾+河源+阳江+清远+东莞+中山+潮州+揭阳+云浮+济南+青岛+淄博+枣庄+东营+烟台+潍坊+济宁+泰安+威海+日照+临沂+德州+聊城+滨州+菏泽+沈阳+大连+鞍山+抚顺+本溪+丹东+锦州+营口+阜新+辽阳+盘锦+铁岭+朝阳+葫芦岛)) Filter:Date:*-:2000-2010 | 500 |
| WanFang | 主题:(流感or流行性感冒or甲流or乙流)not主题:(禽流感or猪流感or流感嗜血杆菌)and主题:(阳性率or病毒学or实验室诊断or实验室确诊)and(全部:(黑龙江or广东or山东or辽宁or上海)or全部:(哈尔滨or齐齐哈尔or鸡西or鹤岗or双鸭山or大庆or伊春or佳木斯or七台河or牡丹江or黑河or绥化or大兴安岭)or全部:(广州or韶关or深圳or珠海or汕头or佛山or江门or湛江or茂名or肇庆or惠州or梅州or汕尾or河源or阳江or清远or东莞or中山or潮州or揭阳or云浮)or全部:(济南or青岛or淄博or枣庄or东营or烟台or潍坊or济宁or泰安or威海or日照or临沂or德州or聊城or滨州or菏泽)or全部:(沈阳or大连or鞍山or抚顺or本溪or丹东or锦州or营口or阜新or辽阳or盘锦or铁岭or朝阳or葫芦岛)) Filter: Date: ^a^-:2000-2010 | 685 |

^a^ Heilongjiang, Guangdong, Shandong, Liaoning, Shanghai

## Table S6. Basic information on the included studies.

| Study | Journal | Language | Study site | Study period | Model method | Mortality measurement | Cause of death |
| --- | --- | --- | --- | --- | --- | --- | --- |
| Cheng, 2022^5^ | Preventive Medicine | Chinese | Zhejiang | 2016-2019 | Distributed lag nonlinear | Influenza-associated excess mortality | AC |
| Yang, 2013^6^ | Chinese Journal of Public Health | Chinese | Harbin, Heilongjiang | 2005/1-2009/12 | Poisson regression | Influenza-associated excess deaths | AC |
| Wu, 2018^7^ | Influenza and Other Respiratory Viruses | English | Beijing | 2007/7-2013/6 | Negative-binomial | Influenza-associated excess mortality | R&C, AC |
| Li, 2015^8^ | International Journal of Virology | Chinese | Dalian, Liaoning | 2000/7-2008/6 | Serfling regression, Negative-binomial | Influenza-associated excess mortality | R&C, AC |
| Chen, 2010^9^ | Disease Surveillance | Chinese | Qingdao, Shandong | 2001/7-2008/6 | Serfling regression | Influenza-associated excess mortality | R&C, AC |
| Hu, 2008^10^ | Anhui Medical University | Chinese | Shanghai | 2000/4-2007/3 | Rate difference | Influenza-associated excess mortality | R&C, AC |
| Lao, 2016^11^ | Preventive Medicine | Chinese | Ningbo, Zhejiang | 2010/1-2014/12 | Rate difference | Influenza-associated excess mortality | R&C, AC |
| Xiang, 2014^12^ | Kunming Medical University | Chinese | Kunming, Yunnan | 2008/4-2013/3 | Serfling regression | Influenza-associated excess mortality | R&C, AC |
| Huang, 2017^13^ | Chinese Journal of Disease Control & Prevention | Chinese | Shenzhen, Guangdong | 2013/4-2016/3 | Serfling regression | Influenza-associated excess mortality | R&C, AC |
| Wong, 2012^14^ | Hong Kong Medical Journal | English | Guangzhou, Guangdong | 2004/1-2006/12 | Poisson regression | Influenza-associated excess mortality | R&C, AC |
| Wang, 2014^15^ | Vaccine | English | Guangzhou, Guangdong | 2010/1-2012/12 | Negative-binomial | Influenza-associated excess deaths | R&C, AC |

R&C, Respiratory and circulatory diseases; AC, All-cause diseases.

## Table S7. Literature scoring results of the included studies.

| **Study** | **Publication** | | **Data reliability** | | **Regional representativeness** | | **Modeling method** | | **Result precision** | | **Total score** |
| --- | --- | --- | --- | --- | --- | --- | --- | --- | --- | --- | --- |
|  | **Score** | **Justification** | **Score** | **Justification** | **Score** | **Justification** | **Score** | **Justification** | **Score** | **Justification** |  |
| Cheng, 2022^5^ | 1 | Preventive Medicine, 2022 | 1 | Weekly mortality: Zhejiang Mortality Register and Surveillance System; Influenza virology: Zhejiang influenza surveillance system; Population: Statistics Bureau of Zhejiang; Daily average temperature: China Meteorological Science Data Sharing Service Network | 1 | Time scales: 4 seasons (week 1~);  Sentinel hospitals: Not needed. Deaths: All deaths registered in Zhejiang.  The results can be well representative of population in Zhejiang. | 1 | Distributed lag nonlinear model: Proportion of ILI cases (ILI%), positive rate of influenza and daily average temperature. | 1 | Excess mortality, deaths, CIs are adequately described. | 5 |
| Yang, 2013^6^ | 1 | Chinese Journal of Public Health  , 2013 | 1 | Weekly mortality: Heilongjiang and Harbin Mortality Register and Surveillance System; Influenza virology: National influenza surveillance system; Meteorology: Meteorological Bureau; Population: China CDC. | 1 | Time scales: 5 seasons (week 1~). Sentinel hospitals: NA, national. Deaths: All deaths in Harbin. The results can be well representative of population in Harbin. | 1 | Poisson regression model: linear and nonlinear time trends, seasonal variation in mortality, and long-term trends in changes in influenza-related deaths, weekly temperature and humidity. | 0.5 | Excess deaths are described but without any CIs. | 4.5 |
| Wu, 2018^7^ | 1 | Influenza Other Respi Viruses, 2018 | 1 | Mortality: Beijing Mortality Register and Surveillance System; Influenza virology: BJCDC. | 1 | Time scales: 6 seasons (week 27~). Sentinel hospitals: 14~23, municipal or national. Deaths: All deaths among registered Beijing population. The results can be well representative of population in Beijing. | 1 | Negative binomial model: weekly mortality, influenza virology data, weeks, cyclical pattern in deaths. | 1 | Excess mortality, deaths, CIs are adequately described. | 5 |
| Li, 2015^8^ | 1 | International Journal of Virology  , 2015 | 1 | Weekly mortality: Dalian Mortality Register and Surveillance System; Influenza virology: National influenza surveillance system; Population: Health Bureau of Dalian. | 0.5 | Time scales: 3 seasons (week 44~12). Sentinel hospitals: Not needed. Deaths: All deaths from 5 districts in Dalian, covered more than 67% of the total population. The number of weekly tested samples in Dalian is small. Data from all the monitoring sites in northern China in the national influenza surveillance network were collected to represent that in Dalian. | 1 | Serfling regression model: linear and nonlinear time trends, seasonal variation in mortality, and long-term trends in deaths.  Negative binomial model: weekly mortality, influenza virology data, weeks, cyclical pattern in deaths. | 1 | Excess mortality, deaths and CIs are adequately described. | 4.5 |
| Chen, 2010^9^ | 1 | Disease Surveillance  , 2010 | 1 | Weekly mortality: Qingdao Mortality Register and Surveillance System; Population: Qingdao Public Security Bureau. | 1 | Time scales: 7 seasons (month 7~). Sentinel hospitals: Not needed. Deaths: All deaths in Qingdao. The results can be well representative of population in Qingdao. | 1 | Serfling regression model: linear and nonlinear time trends, seasonal variation in mortality, and long-term trends in deaths. | 1 | Excess mortality, deaths and CIs are adequately described. | 5 |
| Hu, 2008^10^ | 0.5 | Anhui Medical University  , 2008 | 1 | Weekly mortality: Shanghai CDC Mortality Register and Surveillance System; Influenza virology: National influenza surveillance system; Population: Shanghai Public Security Bureau. | 1 | Time scales: 7 seasons (week 14~). Sentinel hospitals: NA, national. Deaths: All deaths in Shanghai. The results can be well representative of population in Shanghai. | 0.5 | Rate difference model. Only influenza virus activity data and mortality data are included in the model, without considering meteorology, long-term trends, etc. | 0.5 | Excess mortality and deaths are described but without any CIs. | 3.5 |
| Lao, 2016^11^ | 1 | Preventive Medicine  , 2016 | 1 | Weekly mortality: Ningbo CDC Mortality Register and Surveillance System; Influenza virology: National influenza surveillance system; Population: Ningbo Public Security Bureau. | 1 | Time scales: 5 seasons (week 1~). Sentinel hospitals: NA, national. Deaths: All deaths in Ningbo. The results can be well representative of population in Ningbo. | 0.5 | Rate difference model. Only influenza virus activity data and mortality data are included in the model, without considering meteorology, long-term trends, etc. | 0.5 | Excess mortality and deaths are described but without any CIs. | 4 |
| Xiang, 2014^12^ | 0.5 | Kunming Medical University  , 2014 | 1 | Weekly mortality: Chinese Mortality Register and Surveillance System; Influenza virology: Influenza surveillance system in Kunming; Population: Kunming Health Statistics Yearbook and China Disease Surveillance Information Report Management System. | 1 | Time scales: 5 seasons (week 14~). Sentinel hospitals: Not needed. Deaths: All deaths in Kunming. The results can be well representative of population in Kunming. | 1 | Serfling regression model: linear and nonlinear time trends, seasonal trends in mortality, and long-term trends in changes in influenza-related deaths | 0.5 | Excess mortality and deaths are described and with CIs. However, the CIs for 2008-2009 were miscalculated. | 4 |
| Huang, 2017^13^ | 1 | Chinese Journal of Disease Control & Prevention  , 2017 | 1 | Weekly mortality: Shenzhen CDC; Population: Statistics Bureau of Shenzhen. | 1 | Time scales: 3 seasons (week 14~). Sentinel hospitals: Not needed. Deaths: All deaths cases in Shenzhen. The results can be well representative of population in Shenzhen. | 1 | Serfling regression model: linear and nonlinear time trends, seasonal variation in mortality, and long-term trends in deaths. | 1 | Excess mortality, deaths and CIs are adequately described. | 5 |
| Wong, 2012^14^ | 1 | Hong Kong Medical Journal, 2012 | 0.5 | Influenza virology: Surveillance networks for influenza in Guangzhou. Other data sources are not clearly indicated. Influenza season was not defined. | 0.5 | Time scales: 3 seasons (week 1~). Sentinel hospitals: NA. Deaths: NA. The evidence is not sufficient. | 1 | Poisson regression model: weekly mortality, influenza virology data, long-term trends and seasonal variations in mortality, temperature and humidity. | 1 | Excess mortality and CIs are adequately described. | 4 |
| Wang, 2014^15^ | 1 | Vaccine, 2014 | 1 | Weekly mortality data: Guangzhou CDC; Influenza virological data: Influenza surveillance system in Guangzhou. | 1 | Time scales: 3 seasons (week 1~). Sentinel hospitals: 4, national, covered more than 70% ILI outpatients in Guangzhou. Deaths: All deaths cases in Guangzhou. The results can be well representative of population in Guangdong. | 1 | Negative binomial model. Models included virology surveillance time series data, weekly deaths, linear and nonlinear time trends, annual fluctuations in deaths. | 1 | Excess mortality and CIs are adequately described. | 5 |

## Table S8. Selected covariates for the next modeling process.

| **Category** | **Covariates** | |
| --- | --- | --- |
|  | **R&C** | **AC** |
| Demography | Dem_rural,  Dem_rural.prop | Dem_rural,  Dem_rural.prop |
| Health resources | - | Res_beds.per.k |
| Medical services | Ser_outpatient,  Ser_inpatient | Ser_outpatient,  Ser_inpatient |
| Influenza virology data | Rate_B  Rate_all.65 | Rate_all  Rate_B  Rate_all.65  Rate_B.65 |

R&C, Respiratory and circulatory diseases; AC, All-cause diseases.

## Table S9. The results of cross validation of the main analysis and sensitivity analyses in influenza-associated excess respiratory and circulatory among older adults aged 65 years and above.

|  | **RMSE (per 100,000 persons)** | **R^2^** | **MAE (per 100,000 persons)** |
| --- | --- | --- | --- |
| Main analysis | 58.4075 | 0.6155 | 48.6074 |
| Sensitivity analysis 1 | 67.2785 | 0.4935 | 55.6665 |
| Sensitivity analysis 2 | 64.8172 | - | 47.1188 |

RMSE, Root mean squared error; R^2^, R squared; MAE, Mean absolute error.

## Table S10. The results of cross validation of the main analysis and sensitivity analyses in influenza-associated excess all-cause among older adults aged 65 years and above.

|  | **RMSE(per 100,000 persons)** | **R^2^** | **MAE (per 100,000 persons)** |
| --- | --- | --- | --- |
| Main analysis | 72.0113 | 0.6400 | 61.6753 |
| Sensitivity analysis 1 | 80.1284 | 0.4993 | 66.7025 |
| Sensitivity analysis 2 | 79.8968 | - | 56.7534 |

RMSE, Root mean squared error; R^2^, R squared; MAE, Mean absolute error.

## Table S11. Comparison of estimates of annual influenza-associated excess respiratory and circulatory and all-cause mortality (per 100,000) among older adults aged 65 years and above after the 2009 pandemic.

| **Author** | **Study site** | **Study period** | **Model method** | **Excess mortality rate** | |
| --- | --- | --- | --- | --- | --- |
|  |  |  |  | **R&C** | **AC** |
| Narayan VV, et al.^16^ | India | 2010-2013 | Negative binomial model | 122·9 | - |
| Czaja CA, et al.^17^ | Colorado, America | 2007-2016 | Negative binomial model | 46.8 | - |
| Lytras T, et al.^18^ | Greece | 2013-2017 | Distributed-lag nonlinear model | - | 100.5 |
| Nielsen J, et al.^19^ | [Europe](javascript:;) | 2012-2018 | FluMOMO model | - | 101.9 |
| Nielsen J, et al.^20^ | Denmark | 2010-2016 | FluMOMO model | - | 95.9 |
| Wong YJ, et al.^21^ | Hong Kong, China | 2009-2016 | Linear model | - | 128.6 |

R&C, Respiratory and circulatory diseases; AC, All-cause diseases.

# Supplementary Figures

##
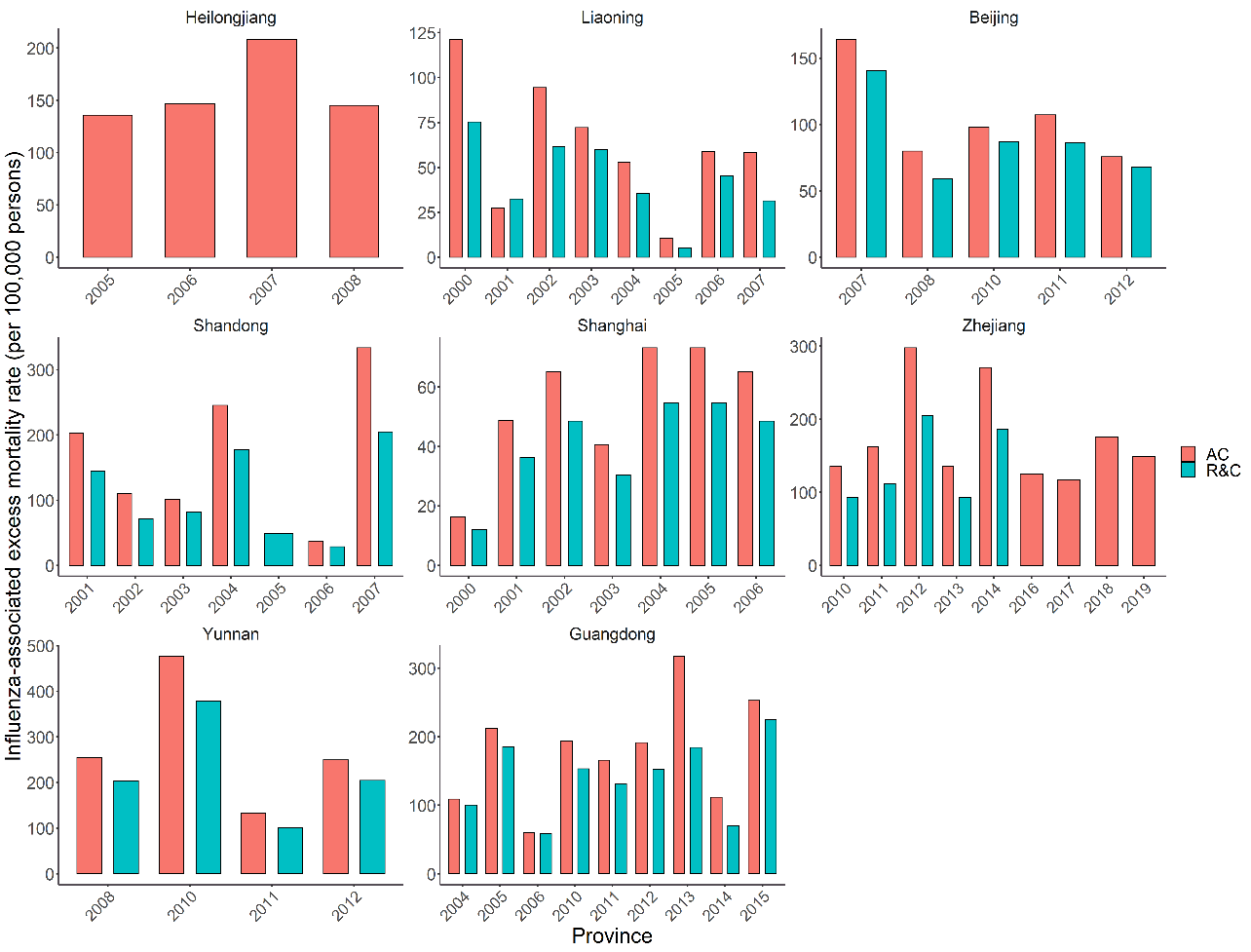
Figure S1. Influenza-associated excess respiratory and circulatory (R&C) and all-cause (AC) mortality among older adults aged 65 years and above by province and by year from the included studies.

##

Figure S2. Estimated annual average influenza-associated excess respiratory and circulatory and all-cause mortality among older adults aged 65 years and above in 30 provinces in China, 2011-2016.

Sensitivity analysis 3 (purple) indicates the estimated results obtained removing extremely low influenza-associated excess mortality data. Dots indicate the point estimates of excess mortality rates. Lines indicate the 95% CIs.

##
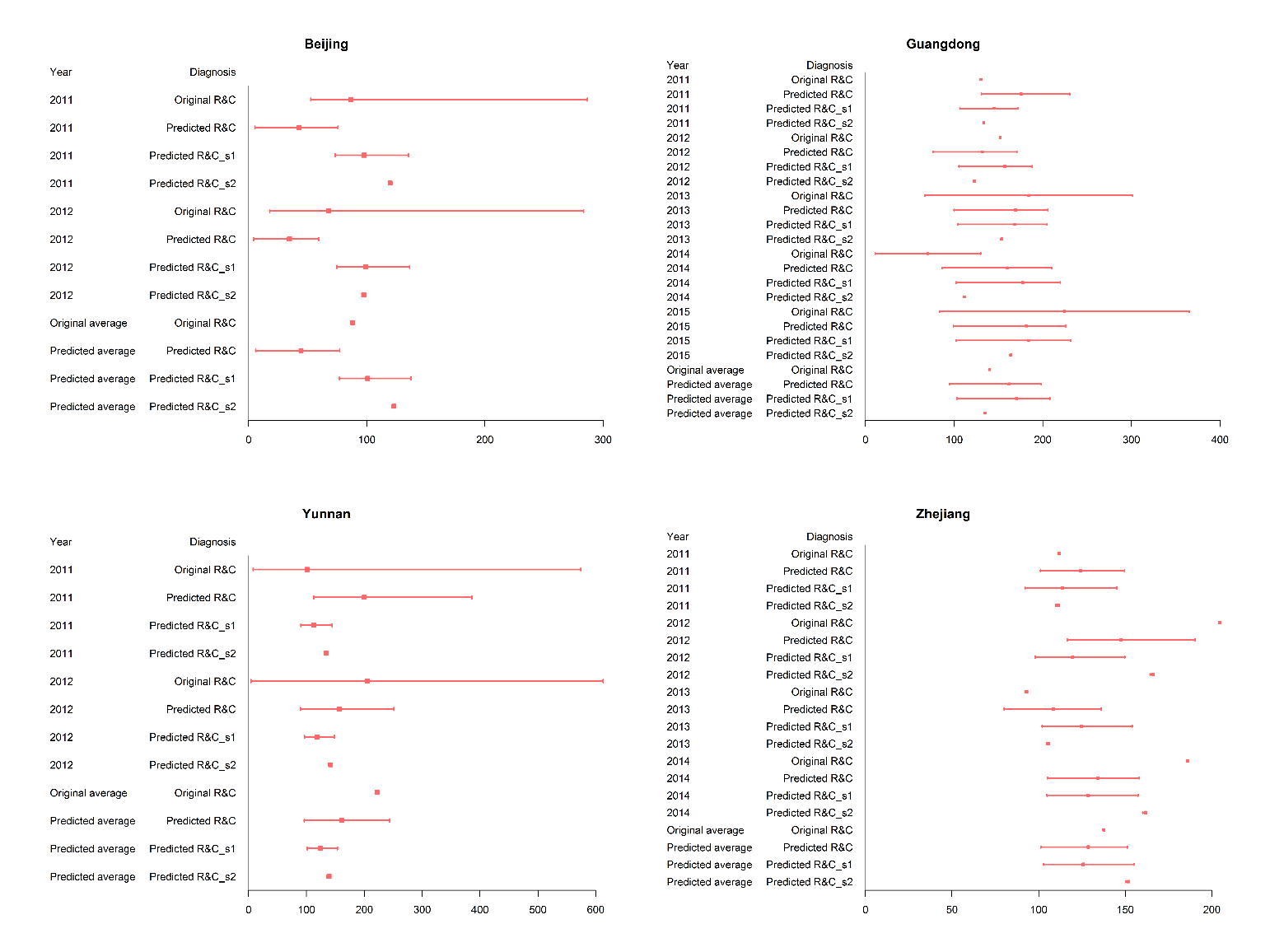
Figure S3. Internal comparison of influenza-associated excess respiratory and circulatory (R&C) mortality among older adults aged 65 years and above between the estimates and published results in available provinces.

Original R&C indicates the original excess R&C mortality from the published literatures included in our study; Predicated R&C indicates the estimated excess R&C mortality in our main analysis; Predicated R&C_s1 indicates the estimated excess R&C mortality in sensitivity analysis 1 using higher-quality data; Predicated R&C_s2 indicates the estimated excess R&C mortality in sensitivity analysis 2 using the random forest approach.

##
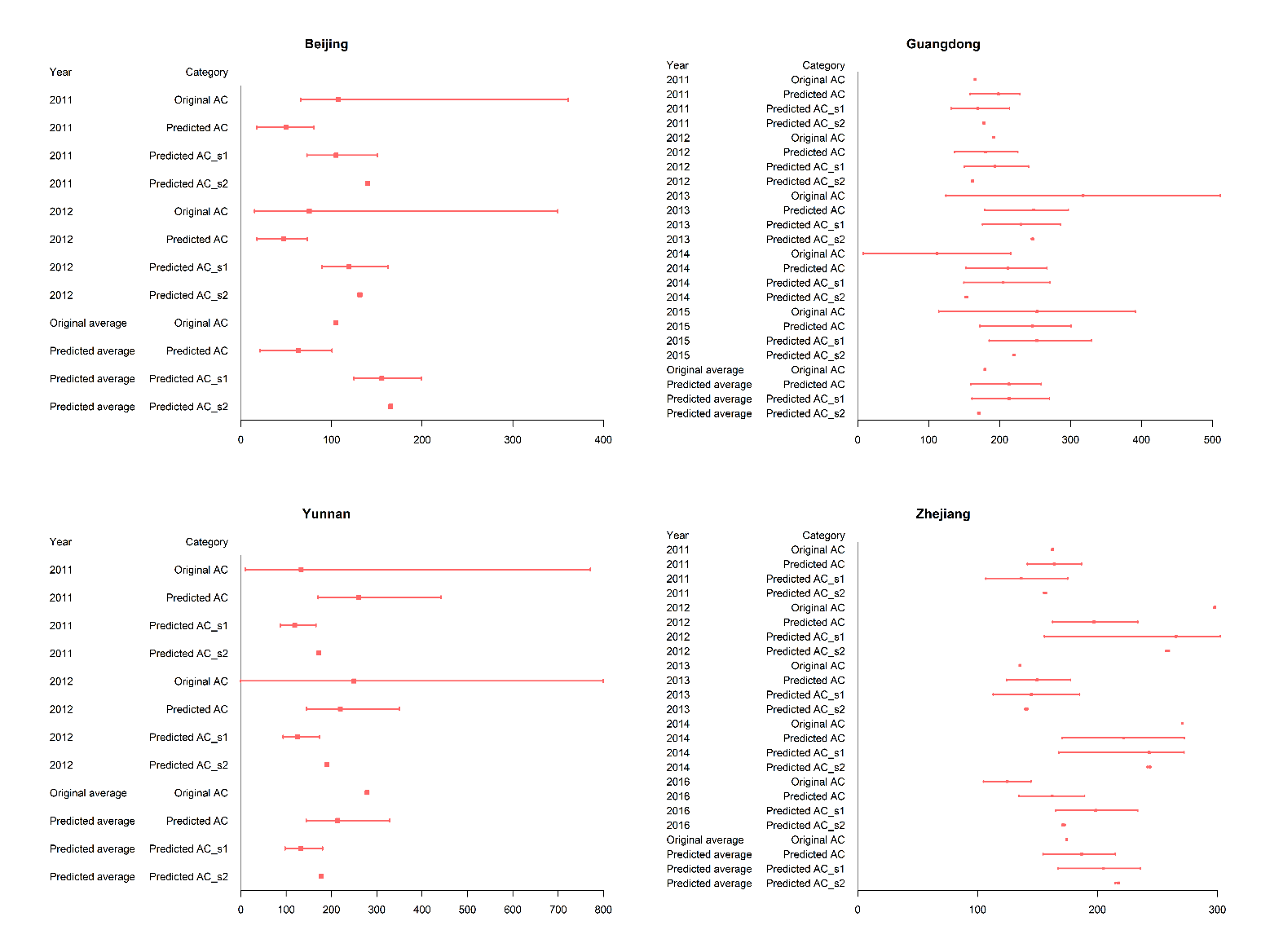
Figure S4. Internal comparison of influenza-associated excess all-cause (AC) mortality among older adults aged 65 years and above between the estimates and published results in available provinces.

Original AC indicates the original excess AC mortality from the published literatures included in our study; Predicated AC indicates the estimated excess AC mortality in our main analysis; Predicated AC_s1 indicates the estimated excess AC mortality in sensitivity analysis 1 using higher-quality data; Predicated AC_s2 indicates the estimated excess AC mortality in sensitivity analysis 2 using the random forest approach.

##
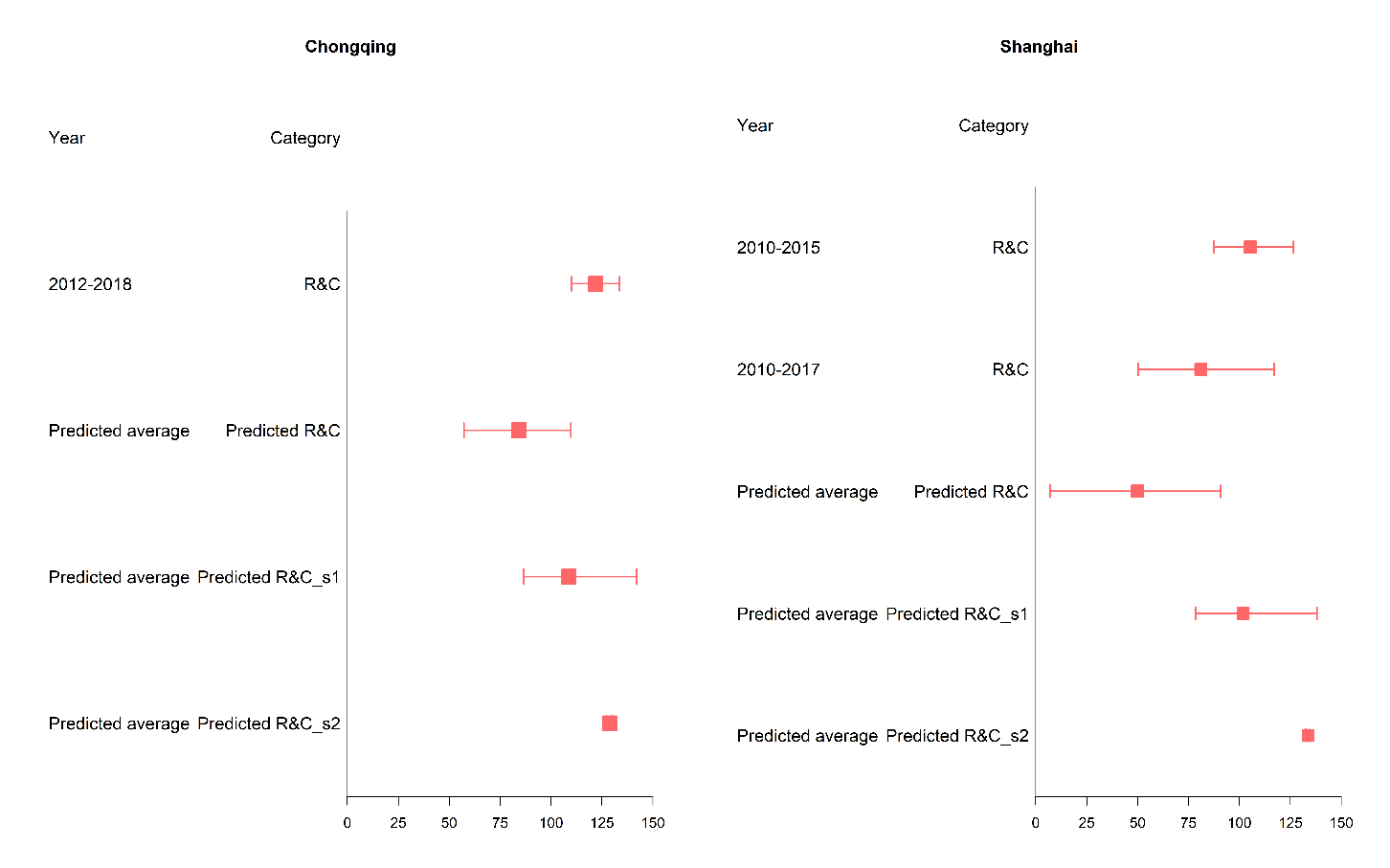
Figure S5. External comparison of influenza-associated excess respiratory and circulatory (R&C) mortality among older adults aged 65 years and above between the estimates and published results in available cities.

R&C indicates the average excess R&C mortality during the research periods; Predicated R&C indicates the average estimated excess R&C mortality from 2011-2016 in our main analysis; Predicated R&C_s1 indicates the average estimated excess R&C mortality from 2011-2016 in sensitivity analysis 1 using higher-quality data; Predicated R&C_s2 indicates the average estimated excess R&C mortality from 2011-2016 in sensitivity analysis 2 using the random forest approach.

##
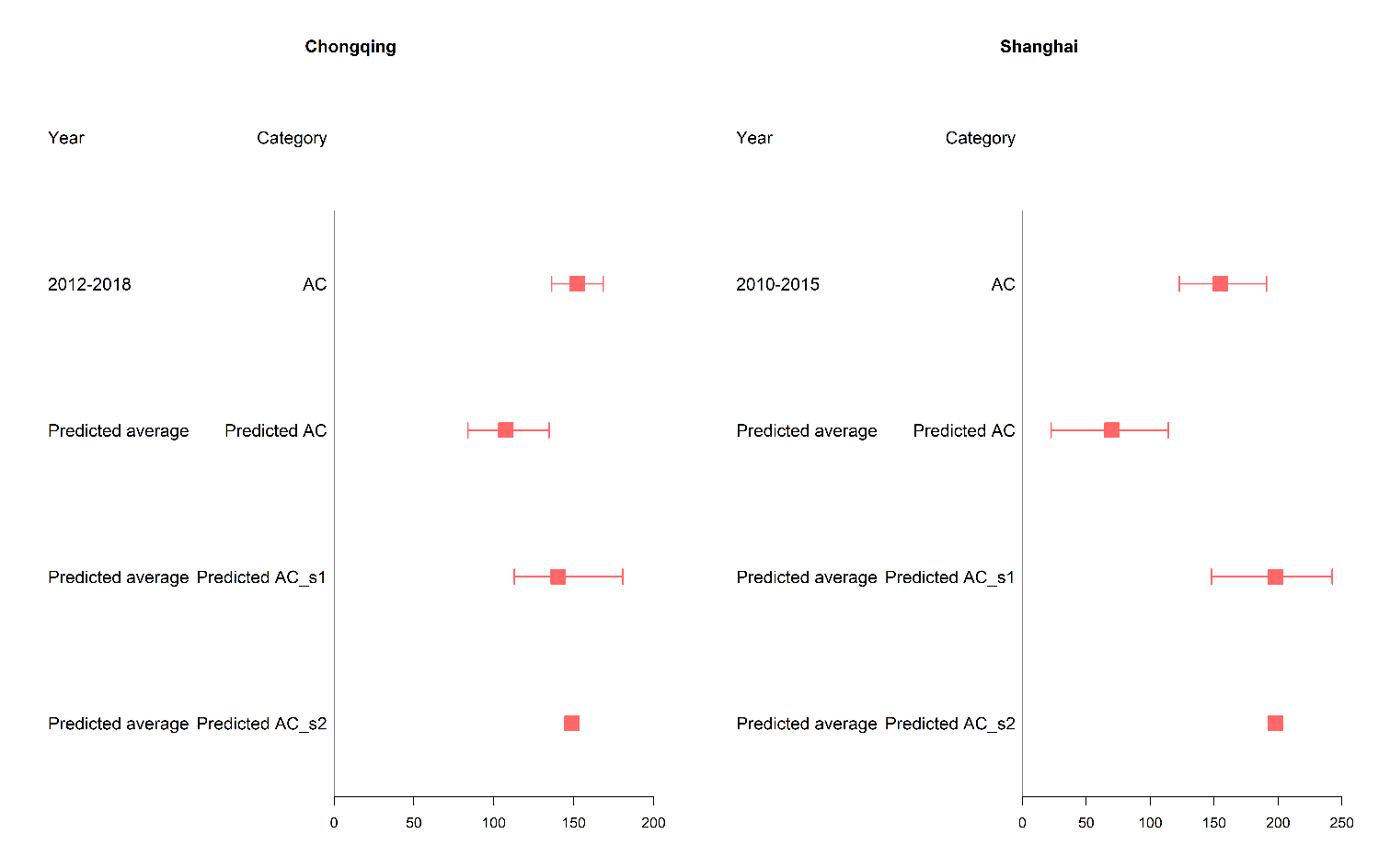
Figure S6. External comparison of influenza-associated excess all-cause (AC) mortality among older adults aged 65 years and above between the estimates and published results in available cities.

AC indicates the average excess AC mortality during the research periods; Predicated AC indicates the average estimated excess AC mortality from 2011-2016 in our main analysis; Predicated AC_s1 indicates the average estimated excess AC mortality from 2011-2016 in sensitivity analysis 1 using higher-quality data; Predicated AC_s2 indicates the average estimated excess AC mortality from 2011-2016 in sensitivity analysis 2 using the random forest approach.

##
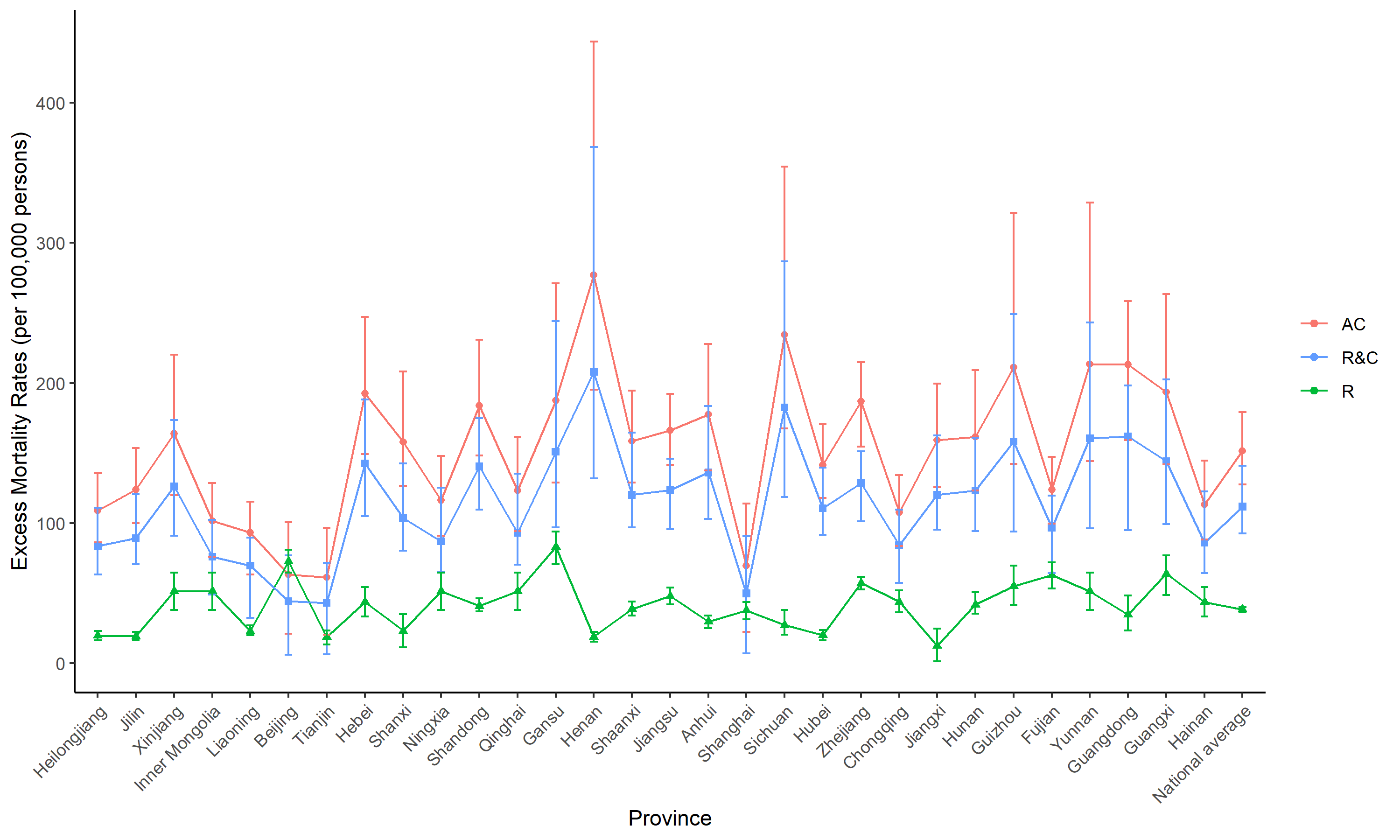
Figure S7. Comparison of the provincial-level variation trend between our estimates of influenza-associated excess respiratory and circulatory (R&C) and all-cause (AC) mortality in the main analysis and the estimates of influenza-associated excess respiratory (R) mortality published previously

References

1. Manning WG, Mullahy J. Estimating log models: to transform or not to transform? *J Health Econ.* 2001;20(4):461-494.

2. Cavanaugh JE. Unifying the derivations for the Akaike and corrected Akaike information criteria. *Statistics & Probability Letters.* 1997;33(2):201-208.

3. Burnham KP, Anderson DR. Model Selection and Multi‐Model Inference: A Practical Information-Theoretic Approach. *New York: Springer;* 2002. 66 p.

4. Liaw A, Wiener M. Classification and regression by randomForest. *R news.* 2002;2(3):18-22.

5. Cheng W, Zhou XY, Yu Z, et al. Influenza-associated excess mortality in Zhejiang Province from 2016 to 2019. *Preventive Medicine.* 2022;34(9):5.

6. Yang L, Zhou H, Che M, et al. Influenza-related excess mortality in Harbin city,2005-2009. *Chinese Journal of Public Health.* 2013.

7. Wu S, Wei Z, Greene CM, et al. Mortality burden from seasonal influenza and 2009 H1N1 pandemic influenza in Beijing, China, 2007-2013. *Influenza and Other Respiratory Viruses.* 2018;12(1):88-97.

8. Li S, Lin H, Feng L, Yu H. Estimates of influenza-associated excess mortality by two regression models in Dalian city during 1991-2008. *International Journal of Virology.* 2015;22(03):172-179.

9. Chen X, Jiang Y, Wang S, Feng L, Zhang K, Zhang Q. Estimate of excess mortality attributed to influenza in Qingdao,2001-2008. *Disease Surveillance.* 2010.

10. Hu A. *Study on excess mortality of seasonal influenza in Shanghai from 2000 to 2006* [D], Anhui Medical university; 2008.

11. Lao X, Jiao S, Ji W, Yi B. An analysis on the influenza -related excess mortality in Ningbo City. *Preventive Medicine.* 2016;28(10):1010-1013+1018.

12. Xiang Y. *Epidemiologic characteristics of influenza in Kunming from 2008-2011 and preliminary application of new technology for analyzing* [D], Kunming Medical University; 2014.

13. Huang Z, Liu X, Wu Y, et al. Application of Serfling cyclical regression model in the estimation of influenza-associated excess mortality in Shenzhen. *Chinese Journal of Disease Control & Prevention.* 2017;21:11 [Article in Chinese].

14. Wong CM, Peiris JS, Yang L, et al. Effect of influenza on cardiorespiratory and all-cause mortality in Hong Kong, Singapore and Guangzhou. *Hong Kong Med J.* 2012;18 Suppl 2:8-11.

15. Wang H, Fu C, Li K, et al. Influenza associated mortality in Southern China, 2010-2012. *Vaccine.* 2014;32(8):973-978.

16. Narayan VV, Iuliano AD, Roguski K, et al. Burden of influenza-associated respiratory and circulatory mortality in India, 2010-2013. *J Glob Health.* 2020;10(1):010402.

17. Czaja CA, Miller L, Colborn K, et al. State-level estimates of excess hospitalizations and deaths associated with influenza. *Influenza Other Respir Viruses.* 2020;14(2):111-121.

18. Lytras T, Pantavou K, Mouratidou E, Tsiodras S. Mortality attributable to seasonal influenza in Greece, 2013 to 2017: variation by type/subtype and age, and a possible harvesting effect. *Euro Surveill.* 2019;24(14).

19. Nielsen J, Vestergaard LS, Richter L, et al. European all-cause excess and influenza-attributable mortality in the 2017/18 season: should the burden of influenza B be reconsidered? *Clin Microbiol Infect.* 2019;25(10):1266-1276.

20. Nielsen J, Krause TG, Molbak K. Influenza-associated mortality determined from all-cause mortality, Denmark 2010/11-2016/17: The FluMOMO model. *Influenza Other Respir Viruses.* 2018;12(5):591-604.

21. Wong JY, Goldstein E, Fang VJ, Cowling BJ, Wu P. Real-time estimation of the influenza-associated excess mortality in Hong Kong. *Epidemiol Infect.* 2019;147:e217.
